# DadY (PA5303) is required for fitness of *Pseudomonas aeruginosa* when growth is dependent on alanine catabolism

**DOI:** 10.15698/mic2022.12.788

**Published:** 2022-11-22

**Authors:** Ronnie L. Fulton, Diana M. Downs

**Affiliations:** 1 Department of Microbiology, University of Georgia, Athens, GA 30602-2605.

**Keywords:** PA5303, D-alanine, Rid, imine, DadA

## Abstract

*Pseudomonas aeruginosa* inhabits diverse environmental niches that can have varying nutrient composition. The ubiquity of this organism is facilitated by a metabolic strategy that preferentially utilizes low-energy, non-fermentable organic acids, such as amino acids, rather than the high-energy sugars preferred by many other microbes. The amino acid alanine is among the preferred substrates of *P. aeruginosa*. The *dad* locus encodes the constituents of the alanine catabolic pathway of *P. aeruginosa.* Physiological roles for DadR (AsnC-type transcriptional activator), DadX (alanine racemase), and DadA (D-amino acid dehydrogenase) have been defined in this pathway. An additional protein, PA5303, is encoded in the *dad* locus in *P. aeruginosa*. PA5303 is a member of the ubiquitous Rid protein superfamily and is designated DadY based on the data presented herein. Despite its conservation in numerous *Pseudomonas* species and membership in the Rid superfamily, no physiological function has been assigned to DadY. In the present study, we demonstrate that DadA releases imino-alanine that can be deaminated by DadY *in vitro*. While DadY was not required for alanine catabolism in monoculture, *dadY* mutants had a dramatic fitness defect in competition with wild-type *P. aeruginosa* when alanine served as the sole carbon or nitrogen source. The data presented herein support a model in which DadY facilitates flux through the alanine catabolic pathway by removing the imine intermediate generated by DadA. Functional characterization of DadY contributes to our understanding of the role of the broadly conserved Rid family members.

## INTRODUCTION

Amino acids are abundant in nature and serve as structural components of peptides and cell walls, intra- and extra-cellular signaling molecules, as well as nutrient sources for microbial growth. Utilization of amino acids for carbon and nitrogen is important for the proliferation of bacteria in several natural environments including soil, aquatic, and marine niches [1]. Additionally, amino acid catabolism can facilitate bacterial association with animal or plant hosts [2–4]. As such, the elucidation of how bacteria utilize amino acids is foundational to an understanding of their ecology, industrial or agricultural applications, and clinical significance.

*Pseudomonas* species are metabolic generalists that can grow on a wide variety of substrates and inhabit diverse ecological niches. *Pseudomonas aeruginosa* is perhaps the best studied member of the genus due to its clinical significance. The importance of understanding the metabolism and physiology of this organism is reinforced by its ability to influence the microbial composition of its environment by participating in mutualistic relationships and edging-out competitors [5–10]. Like other *Pseudomonas* species, *P. aeruginosa*, has adopted a metabolic strategy in which low energy, non-fermentable organic acids, such as tricarboxylic acid (TCA) cycle intermediates and amino acids (particularly Asn, Asp, Gln, Glu and Ala) are preferentially utilized over high energy sugars like glucose [11–14]. This strategy provides *P. aeruginosa* an advantage over competitors in environments where amino acids, and other organic acids, are the primary source of nutrients.

**Figure 1:**
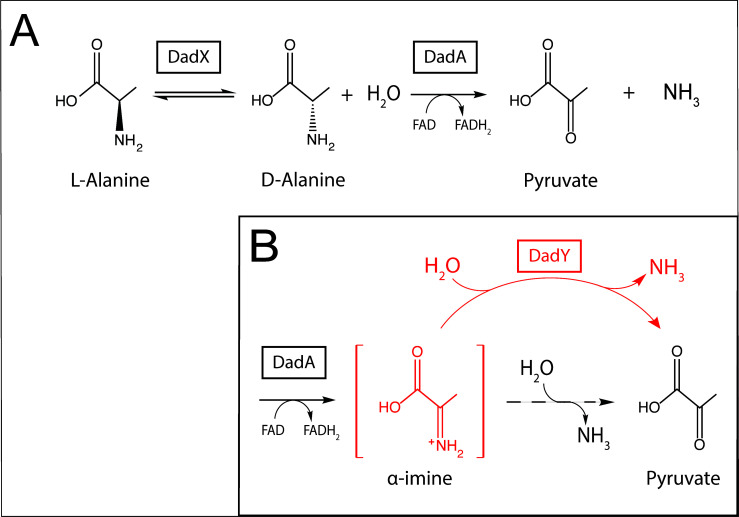
FIGURE 1: Alanine catabolism in *P. aeruginosa*. **(A)** In the established pathway for alanine catabolism in *P. aeruginosa*, L-alanine is converted to D-alanine by the DadX racemase. The FAD-dependent D-amino acid dehydrogenase, DadA, then oxidizes D-alanine which results in the generation of pyruvate and ammonia. **(B)** The proposed role for DadY in the catabolism of alanine by *P. aeruginosa* is shown. After oxidation of D-alanine at the alpha carbon, DadA releases imino-alanine into the cellular milieu (red, brackets). This α-imine can be deaminated either spontaneously by solvent water (dashed arrow) or by DadY (red), both of which generate the final products, pyruvate and ammonia.

Alanine is among the preferred amino acid substrates for *P. aeruginosa*. Catabolism and homeostasis of this amino acid have been implicated in virulence, gene regulation, and mechanical properties of the cell [15–17]. The *dad* locus in *P. aeruginosa*, regulated by DadR (AsnC-type regulator), encodes DadX (PLP-dependent alanine racemase, EC 5.1.1.1), DadA (FAD-dependent D-amino acid dehydrogenase, EC 1.4.5.1), and PA5303, a protein of unknown function. Based on the data presented herein, PA5303 is designated DadY throughout. DadA and DadX have been biochemically characterized and their physiological roles in the catabolism of alanine described [15]. The alanine catabolic pathway that incorporates these activities is schematically represented in **Figure 1A**. DadY belongs to the large, sequence diverse Rid (Reactive intermediate deaminase) superfamily of proteins, which is categorized into eight subfamilies. Within this superfamily, the ancestral RidA subfamily is conserved in all domains of life, while the remaining subfamilies are found only in prokaryotes, primarily bacteria [18]. Rid proteins can also be divided into two groups based on the presence or absence of a key arginine residue that is essential for imine/enamine deaminase activity. The deaminase activity of these proteins was first described for the RidA in *Salmonella enterica* [19]. Proteins in the RidA and Rid1-3 subfamilies, including RutC proteins, contain the relevant arginine residue, and all such proteins that have been assayed to date have some imine/enamine deaminase activity *in vitro* [18–29]. Significantly, the deaminase reaction catalyzed by Rid proteins assessed thus far can also be carried out spontaneously by solvent water, suggesting that these proteins simply accelerate a reaction that can occur in their absence [19–22, 29–31]. Members of the RidA subfamily have been assigned a role in ameliorating 2-aminoacrylate stress in a paradigm that has been well established in several bacterial species, yeast, and higher-order eukaryotes [2, 23–25, 30–34]. The deaminase activity of proteins in the other Rid subfamilies, except for RutC from *Escherichia coli* [22], has not been placed in a physiological context [15, 20, 21, 24, 35]. Proteins belonging to the Rid4-7 subfamilies lack the relevant Arg residue and, as expected, those tested to date lack imine/enamine deaminase activity [18, 20, 21]. Currently, RidA proteins are the only Rid subfamily with members that have a demonstrated physiological role.

DadY belongs to the Rid2 subfamily and, based on the presence of the relevant arginine residue, is predicted to have deaminase activity. In *P. aeruginosa, dadY* is in an operon with *dadA* and *dadX*, which implicates DadY in alanine catabolism. DadA is an FAD-dependent dehydrogenase, the catalytic mechanism of which has been proposed to proceed through an imine intermediate, as demonstrated for other similar dehydrogenases [21, 36–38]. Were this the case, the imine (imino-alanine, or 2-iminopropionate) released by DadA would be a potential substrate for DadY. In this scenario, deaminase activity of DadY would accelerate the rate of formation of the final products of the pathway – pyruvate and ammonia (**Figure 1B**). This study was initiated to test the above scenario and define the physiological role for DadY in *P. aeruginosa*. The data show that DadY improves fitness of *P. aeruginosa* when growth is dependent on alanine catabolism. The results herein emphasize the significance of competitive fitness as a means to detect the subtle, but critical, contributions of various metabolic components to robust cellular physiology.

## RESULTS

### The structure of the *dad* locus varies among phylogenetic groups of *Pseudomonas*

**Figure 2:**
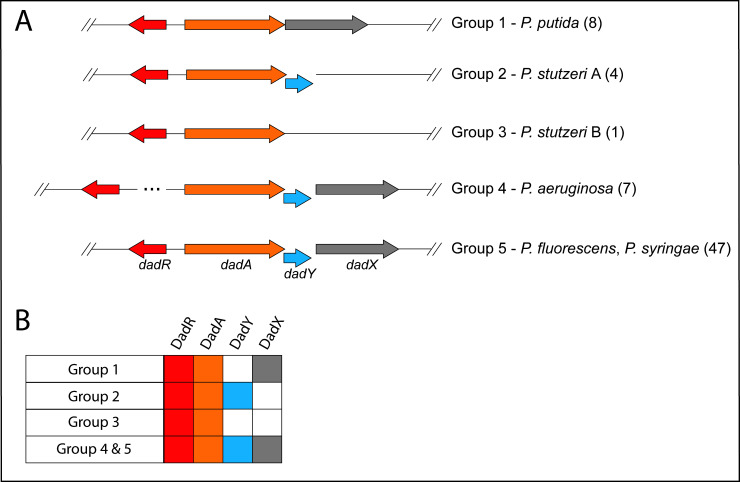
FIGURE 2: Genomic organization of genes in the *dad* locus varies in *Pseudomonas* species. **(A)** Schematic showing the configurations of *dad* loci in *Pseudomonas*. Genomic affinity groups [39–43] were classified based on the organization **(A)** and constituents **(B)** of the *dad* locus. Gene products considered were DadR (AsnC-type regulator, red), DadA (D-amino acid dehydrogenase, yellow), DadX (catabolic alanine racemase, grey), and DadY (Rid2 subfamily protein, blue). The number of species belonging to each group is shown in parentheses. **(B)** Distribution of *dad* genes in *Pseudomonas*. The presence (filled boxes) or absence (empty boxes) of each gene in the *dad* locus is indicated for the groups shown in A.

The *dad* locus of *P. aeruginosa* includes a single gene, *dadA*, that is conserved among all *Pseudomonas* species. The *dadR* gene is also present in all *Pseudomonas* species but is located distally from other *dad* genes in *P. aeruginosa*. *Pseudomonas* species have been previously classified into genomic affinity groups and subgroups based on phylogenetic clustering and sequence similarity [39–43]. The structure of the *dad* locus was assessed in these groups. The phylogenetic groups were placed into one of five categories based on the constituents and organization of *dad* genes (**Figure 2**). This analysis showed that most *Pseudomonas* species have a *dad* locus with four contiguous genes (**Figure 2**; Group 5). Members of the *P. aeruginosa* group similarly encode the four *dad* genes. However, the *P. aeruginosa* group is distinct in that *dadR* is separated from the remaining *dad* genes by three open reading frames (ORFs). Members of the *Pseudomonas putida* group and a single *Pseudomonas stutzeri* species (*P. stutzeri* Group B), lack an identifiable *dadY* in their genome. The remaining species in the *P. stutzeri* group lack a gene encoding DadX, the PLP-dependent racemase that is essential for L-alanine catabolism in *P. aeruginosa* (**Figure 1**) [15]. DadY is a putative imine/enamine deaminase belonging to the Rid superfamily. A gene encoding DadY is present in the *dad* locus of most *Pseudomonas* species (**Figure 2**). Despite its prevalence in the *dad* locus of most *Pseudomonas* species, no function has been described for DadY in the alanine catabolic pathway. A working model suggests that DadY can deaminate the imino-alanine generated by DadA, which will increase flux through the pathway as represented in **Figure 1B** [20].

### DadY is an imine deaminase *in vitro*

**Figure 3:**
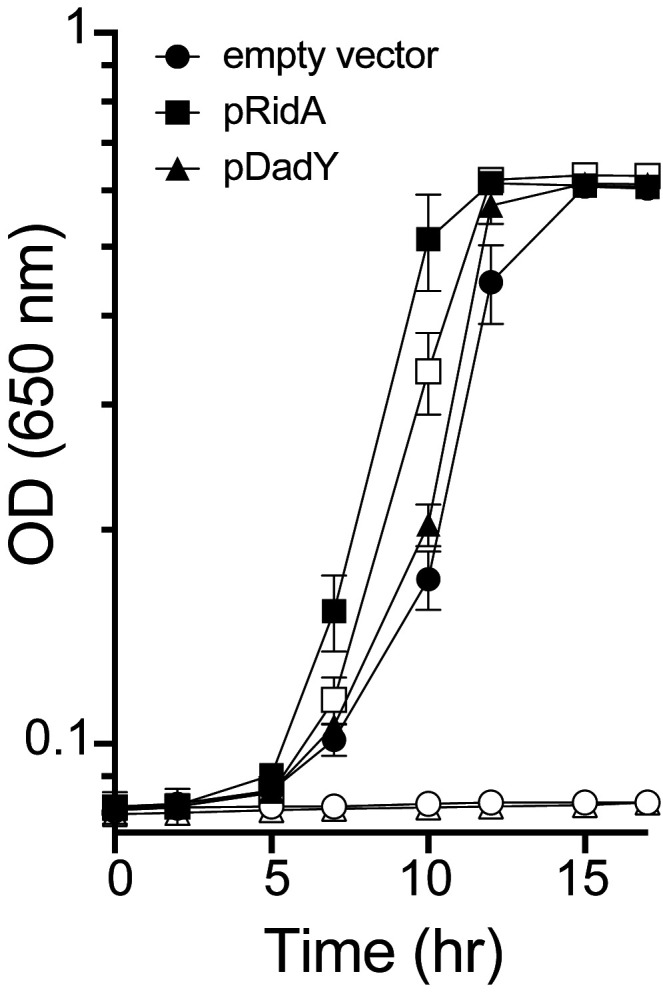
FIGURE 3: DadY fails to complement a *S. enterica ridA* mutant. Growth of a *S. enterica ridA* mutant harboring the control pCV1 vector (circles), pDM1636, encoding RidA from *S. enterica* (squares), or pDM1580, encoding DadY from *P. aeruginosa* (triangles) was monitored in minimal glucose (11 mM) medium without (closed symbols) or with (open symbols) 5 mM L-serine. L-arabinose (0.2%) was present in all media to induce expression of the plasmid-borne gene. Error bars represent standard deviation between three independent biological replicates.

*ridA* mutants of *S. enterica* fail to grow in the presence of serine due to the accumulation of the reactive enamine 2-aminoacrylate (2AA) [19, 30, 44]. These *ridA* mutants provide a means to test other proteins (i.e., Rid proteins) for 2AA deaminase activity *in vivo*, since such activity restores growth to the *ridA* mutant [21, 24, 25]. When provided *in trans,* DadY from *P. aeruginosa* failed to restore growth of a *S. enterica ridA* mutant in the presence of serine, despite the plasmid-borne gene being overexpressed (**Figure 3**). In contrast, _SE_RidA fully restored growth with or without overexpression, as did multiple RidA subfamily members (data not shown) [21, 24]. These data show that the activity of DadY and RidA are not redundant *in vivo*, even though DadY can deaminate 2AA *in vitro* (data not shown). Together these observations raise the question of how deaminase activity of DadY differs from that of RidA *in vitro* and *in vivo*.

The arginine critical for imine/enamine deaminase activity in Rid proteins is present and at position 94 in DadY. The imine deaminase activity of DadY was tested using a standard *in vitro* assay. L-amino acid oxidase (LOX) is an FAD-dependent enzyme that generates imines from a variety of L-amino acid substrates. The resulting imines are released into the solvent where they can i) be quenched by solvent water, ii) react with added semicarbazide to generate a semicarbazone that is detectable spectrophotometrically at 248 nm, or iii) be quenched by a Rid protein with deaminase activity. The rate at which imines are hydrolyzed by solvent water is constant at a given pH but varies for imines derived from different amino acids [45]. In this assay, Rid-mediated deaminase activity is detected as a decrease in semicarbazone formation compared to reactions with LOX and semicarbazide alone. The ability of DadY to deaminate imines generated by LOX from the L-amino acids L-Arg, L-Gln, L-His, L-Leu, L-Met, and L-Phe was determined (**Table 1**). DadY deaminated imines derived from all amino acids tested and had a broader substrate range than either of the RidA proteins. Specifically, neither the RidA from *S. enterica* nor *P. aeruginosa* had significant deaminase activity on imines derived from L-Arg, L-His or L-Phe. Of the substrates the RidA proteins were active with, DadY was significantly more active on imines derived from L-Gln and appeared more active on the imine derived from L-Leu than the *S. enterica* RidA. These data allow the conclusion that DadY is an imine deaminase and has a substrate specificity that differs from RidA proteins.

**Table 1:** DadY is an imine deaminase *in vitro*.

	**Rate of semicarbazone formation (μM/min) with:**			
**LOX Substrate**	**No Rid protein**	**DadY^a^**	**_SE_RidA**	**_PA_RidA**
L-Arg	518 ± 17	28 ± 10^b^	470 ± 58	552 ± 62
L-Gln	193 ± 8	ND	78 ± 13^a^	132 ± 5^a^
L-His	196 ± 10	95 ± 19^b^	196 ± 5	202 ± 17
L-Leu	294 ± 29	20 ± 5	53 ± 5^a^	22 ± 5^a^
L-Met	291 ± 10	45 ± 5	48 ± 5^a^	62 ± 10^a^
L-Phe	232 ± 26	62 ± 5^b^	185 ± 1	188 ± 41

a significant difference in semicarbazone formation (p < 0.05, determined by Tukey's multiple comparisons test) between indicated reactions and those with no Rid protein. Activity of reactions with DadY was significantly different than LOX alone with all amino acids tested.

b significant difference in semicarbazone formation between reactions containing DadY and those with either RidA protein.

Reaction mixes (100 μl) contained potassium pyrophosphate pH 8.7 (50 mM), semicarbazide pH 7.0 (10 mM), bovine liver catalase (24 U), L-amino acid oxidase from *Crotalus adamanteus* (1 μg), and the Rid protein (25 μM, monomeric concentration) indicated. Reactions were initiated by the addition of the indicated substrate (20 mM) and absorbance at 248 nm was monitored over five minutes. The rate of semicarbazone formation was determined using the measured pathlength of each well and the molar extinction coefficient for semicarbazone (ε = 10300 M^-1^ cm^-1^). Data shown are the mean and standard deviation of three technical replicates. ND, no semicarbazone was detected; _SE_RidA, RidA from *S. enterica;*_PA_RidA, RidA from *P. aeruginosa*

### DadA releases an imine substrate for DadY

DadA is a membrane-bound, FAD-dependent D-amino acid dehydrogenase that uses D-alanine as its preferred substrate. DadA cannot use oxygen as a terminal electron acceptor, distinguishing its mechanism from that of LOX and other oxidases [36]. The catalytic mechanism of DadA is thought to proceed via an imine intermediate, as is the case with similar enzymes [21, 36–38]. If generated, the imine intermediate would either remain in the active site of DadA or be released into the solvent prior to the deamination that results in pyruvate and ammonia. Semicarbazide was added to the assay to determine if imino-alanine was released into the solvent after generation by DadA. If released, the imino-alanine would be expected to generate a semicarbazone compound that was detectable spectrophotometrically. When DadA used D-alanine as a substrate and semicarbazide was present, a semicarbazone compound was formed at a rate of 18.3 ± 0.3 μM/min. This result indicated that DadA released an imine product, and thus provided a potential substrate for DadY *in vivo*.

The data above was generally consistent with the proposed role for DadY in the catabolism of alanine (**Figure 1**). DadY was added in the DadA assay described above to determine if this Rid protein could compete with semicarbazide for the released imine. The rate of semicarbazone formation was observed to be nearly four-fold lower in the presence vs. absence of DadY (4.7 ± 0.7 vs 18.3 ± 0.3 μM semicarbazone formed/min, respectively). Results of these assays confirmed that DadY could act on the imine derived from D-alanine that was generated by DadA. In total, the data supported the potential for DadY to contribute to the efficiency of alanine catabolism *in vivo*.

### *P. aeruginosa* catabolizes alanine in the absence of DadY

**Figure 4:**
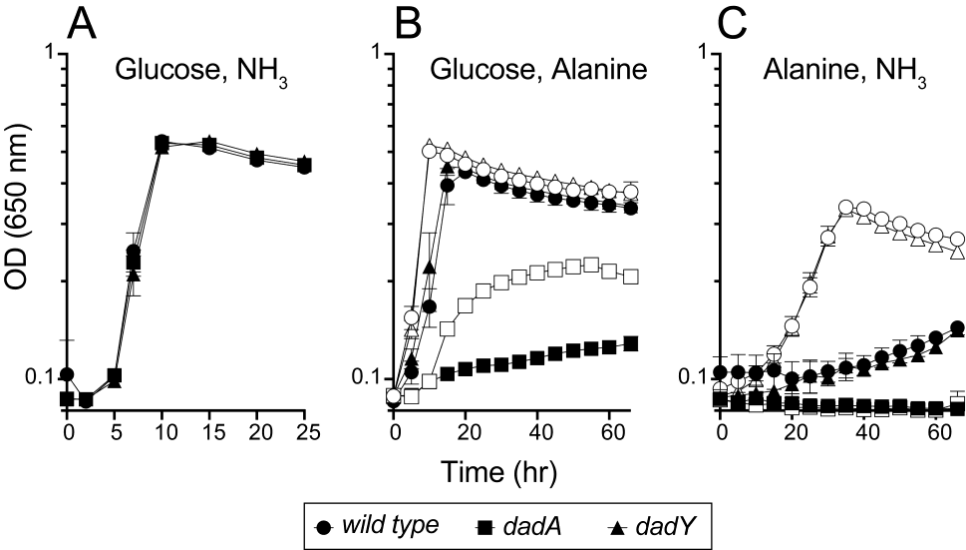
FIGURE 4: DadY is not required for catabolism of alanine by *P. aeruginosa*. *P. aeruginosa* wild-type (circles), *dadA* mutant (squares), and *dadY* mutant (triangles) strains were inoculated into minimal media with the carbon and nitrogen sources as indicated (carbon, nitrogen); **(A)** glucose, ammonia, **(B)** glucose, alanine, and **(C)** alanine, ammonia. D-alanine (closed shapes) or L-alanine (open shapes) was added as indicated as the source of carbon or nitrogen at 20 mM or 5 mM, respectively. When present glucose was at 11 mM and ammonia was at 10 mM. Growth was measured as the change in optical density at 650 nm over time. Error bars represent standard deviation between three independent biological replicates.

**T**he data above implicated DadY in the alanine catabolic pathway and suggested that this protein, like DadA and DadX, could be required for *P. aeruginosa* to use alanine as a nutrient source. Various strains were inoculated into media with D-/L-alanine as the sole source of nitrogen or carbon (**Figure 4**). Growth patterns of wild-type and strains lacking either the *dadA* or *dadY* gene were indistinguishable with glucose and ammonia as the sole source of carbon or nitrogen, respectively. As expected, the *dadA* mutant had a significant growth defect when D- or L-alanine was the sole source of nitrogen or carbon. The defect of the *dadA* mutant was particularly severe when D-alanine served as the sole carbon source, though even wild-type grew poorly in this condition. Poor use of D-alanine as a carbon source was previously attributed to a need to convert D-alanine to the L-alanine required to fully activate transcription of the *dad* operon [15]. Unexpectedly, there was significant growth of the *dadA* mutant when L-alanine was the sole nitrogen source. This result suggests there is another way to generate ammonia from alanine *in vivo*, the mechanism of which was not pursued here. Unlike the varying, but clear, growth defects of a *dadA* mutant, growth of the strain lacking DadY was not significantly different from wild-type in any condition tested (**Figure 4**).

### DadY is required for competitive fitness of *P. aeruginosa*

**Figure 5:**
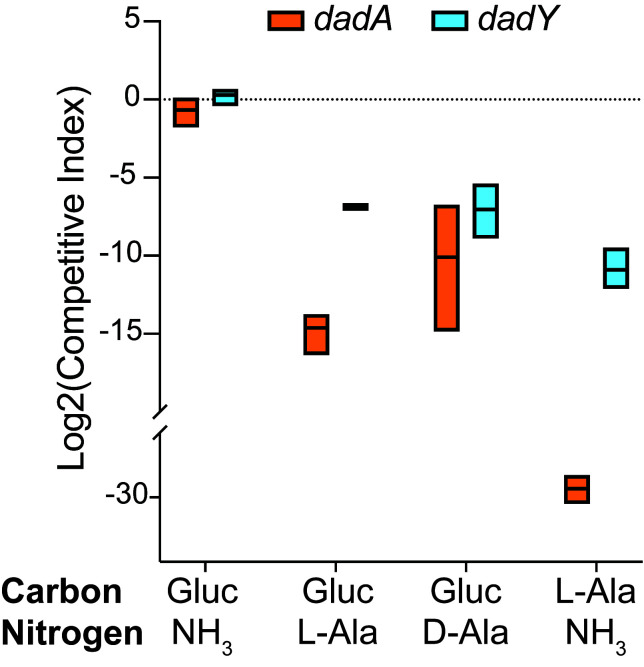
FIGURE 5: *dadA* and *dadY* mutants are outcompeted by wild-type when alanine catabolism is required. Cells of mutant strain DMPA39 (*dadA* Gm^R^, orange) or DMPA40 (*dadY* Gm^R^, blue) were combined with an equal number of DMPA4 (wild-type) cells (final OD_650_ = 0.02) in minimal media with the indicated sources of carbon (11 mM glucose or 20 mM Ala) and nitrogen (10 mM ammonia or 5 mM Ala). Co-cultures were grown to stationary phase at 37°C with shaking. Colony forming units (CFU) were determined by serial dilution and spot plating onto LB Gm30 (*dadA* or *dadY* mutants) and LB without drug (total cells) at the onset of the experiment, and after cultures reached stationary phase. The number of wild-type cells was determined by subtracting the number of each mutant from the total number of cells. The Competitive Index (CI) was calculated as the ratio of mutant to wild-type *P. aeruginosa* after co-incubation, divided by the ratio of mutant to wild-type in the inoculum. Data shown are mean and standard deviation of three independent biological replicates. Experiments were performed three times with similar results.

The biochemical data above showed that DadY can deaminate the imino-alanine generated by DadA, though this reaction can also be mediated by solvent water. Growth data in **Figure 4** suggested that the rate of imine deamination by water was sufficient for growth in monoculture under standard laboratory conditions. One hypothesis for the conservation of DadY is that it contributes to fitness of *P. aeruginosa* when it is competing with other organisms in the environment. This hypothesis was supported by the results of experiments to measure competitive fitness (**Figure 5**). Competitive fitness was determined for strains lacking the appropriate gene (*dadA* or *dadY*) when grown with wild-type *P. aeruginosa* in a variety of media. Importantly, wild-type did not have a competitive advantage over either mutant when grown in a minimal medium with glucose as carbon source and ammonia as nitrogen source. Based on the growth defect in monoculture, it was expected that the *dadA* mutant had a competitive disadvantage in a medium with alanine as sole source of either nitrogen or carbon. However, it was striking that the *dadY* mutants were also outcompeted by wild-type when grown with alanine as the sole source of carbon or nitrogen. In each of the relevant media, the *dadY* mutant had a significant competitive disadvantage, though to a lesser degree than the *dadA* mutant in the same condition. The most significant competitive defects were detected when alanine was provided as the sole source of carbon. This result is not unexpected since the cell requires more carbon than for nitrogen. This means the efficiency of the pathway encoded by the *dad* operon is more critical when it is responsible for generating the carbon molecule (pyruvate) that can enter central metabolism. The *dadY* mutant had a 128-fold decrease in competitive fitness (i.e., the inverse of the Competitive Index – see equation in ”Material and Methods”) when alanine was the nitrogen source versus a 2000-fold decrease when alanine was the carbon source (**Figure 5**). The same bias was observed, to a greater degree, with the *dadA* mutant, which had a fitness defect of 10^9^ and 33,000 when alanine was used as a carbon or nitrogen source, respectively. The more severe defect of the *dadA* mutant compared to the *dadY* mutant in all cases was consistent with growth phenotypes in monoculture. When D-alanine was provided as sole nitrogen source, the competitive fitness of the *dadA* and *dadY* mutants were less defective and more similar to each other than with L-alanine. These data are consistent with the limited ability of wild-type *P. aeruginosa* to grow on D-alanine (**Figure 4**). In total, these data support the hypothesis that DadY is required to accelerate the catabolism of alanine in competitive settings, like the natural environments of *P. aeruginosa.*

## DISCUSSION

Members of the Rid superfamily that have been characterized catalyze reactions *in vitro* that are also mediated spontaneously by solvent water. While imine/enamine deaminase activity can be demonstrated *in vitro,* the role of these enzymes *in vivo* has only been clearly shown for members of the RidA subfamily. Characterization of the RidA protein in *S. enterica* defined the paradigm for 2-aminoacrylate (2AA) stress. 2AA is a reactive enamine that can attack and inactivate target PLP-dependent enzymes. While 2AA can be deaminated spontaneously by water, loss of RidA activity results in the accumulation of 2AA, resulting in global damage that ultimately leads to detectable phenotypes. Importantly, the phenotypes differ between organisms and often require an additional environmental, genetic, or nutritional insult to be detected. Proteins in the Rid1-3 subfamilies are presumed to also have deaminase activity. Genes encoding Rid1-3 proteins are broadly distributed in bacteria and are found in diverse genomic contexts. While numerous Rid1-3 proteins have been assayed for generic imine/enamine deaminase activity *in vitro*, the relevant substrate, and thus physiological role, has not been defined for these proteins. We suggested the lack of phenotypic manifestation reflected a low, yet sufficient, rate of reactivity with water in the cellular milieu [20]. The current study provides the first substantial evidence in support of this general hypothesis. Here, we demonstrate that the Rid2 subfamily protein DadY (PA5303) is required for competitive fitness of *P. aeruginosa* when growth is dependent on the catabolism of alanine.

A role for DadY in alanine catabolism was suggested by its genomic context in *P. aeruginosa*, and other *Pseudomonas* species (**Figure 2**). The putative deaminase activity of this protein family led us to propose the model in **Figure 1**. This model was based on the precedent that FAD-dependent dehydrogenases generate and release imine products, and that Rid family proteins with a key arginine can have imine/enamine deaminase activity. The data herein reconstructed the components of this pathway *in vitro*. However, the simple prediction from this model, that a *dadY* mutant would be defective in catabolizing alanine, was not supported by analyses of growth in monoculture. As noted above, the reaction attributed to DadY in this scheme is also mediated by solvent water, suggesting that DadY might only be needed to accelerate flux through the pathway when the rate of spontaneous deamination by free water is insufficient. In other words, DadY activity could circumvent a kinetic bottleneck caused by reliance on the spontaneous conversion of these imines to the final ketoacid and ammonia products.

*P. aeruginosa*, and most (if not all) microbes, are constantly in competition with one another in their natural environments. Thus, analysis of metabolic efficiency might be more relevant if carried out in a competitive context. This notion is supported by the fact that strains lacking *dadY* were dramatically outcompeted by wild-type when alanine served as sole source of either carbon or nitrogen, despite having no growth defect in monoculture. The results herein support a model for alanine catabolism in which DadY removes imine intermediates released by DadA at a rate greater than that of free water. Such metabolic contributions by DadY appear to be too subtle to elicit a requirement in monoculture but become essential for optimal fitness in a competitive environment. The characterization of DadY activity as supplementary to the metabolic efficiency of *P. aeruginosa* provides a potential framework through which to view the contributions of Rid proteins in general to the metabolic network they participate in. Specifically, it provides the first clear evidence that Rid proteins, at least those belonging to the deaminase group, could serve to fine-tune their respective metabolic pathway by maximizing flux. This acceleration may not be necessary for growth in monoculture but would be advantageous in the natural environments inhabited by competitors. This benefit to fitness would justify the broad representation and conservation of members of this superfamily. Further work is needed to shed light on the separate and/or overlapping activities of the different Rid family members. The possibility that Rid proteins catalyze otherwise spontaneous reactions that are distinct from water mediated deamination must be considered. The existence of Rid superfamily members that are incapable of deamination supports this notion and emphasizes the trove of metabolic knowledge that is likely to be uncovered with a better understanding of the Rid protein superfamily.

## MATERIALS AND METHODS

### Media and chemicals

*P. aeruginosa* and *E. coli* strains were routinely grown in Lysogeny Broth (LB; 10 g/L tryptone, 5 g/L NaCl, 5 g/L yeast extract), and *S. enterica* strains were grown in Nutrient Broth (NB; Difco) as a rich medium. Both bacteria were incubated at 37°C unless otherwise indicated. For protein purification, cells were grown in Terrific Broth (TB; 12 g/L tryptone, 24 g/L yeast extract, 4 ml/L glycerol, 17 mM KH_2_PO_4_, 72 mM K_2_HPO_4_). Solid media contained 15 g/L agar. Ampicillin (Ap) and kanamycin (Km) were added at 150 μg/ml and 50 μg/ml, respectively, for *E. coli*. Gentamicin (Gm) was added at 30-50 μg/ml for *P. aeruginosa* or 20 μg/ml for *E. coli*. Minimal media were M8 salts (12 mM Na_2_HPO_4_, 22 mM KH_2_PO_4_, 1 mM NaCl, 1 mM MgSO_4_) with trace minerals [46], supplemented with indicated sources of carbon (11 mM glucose; 20 mM amino acids) and nitrogen (10 mM NH_4_Cl; 5 mM amino acids). Vogel-Bonner Minimal Medium supplemented with 50 μg/ml gentamicin (VBMM Gm50; 3 g/L trisodium citrate, 2 g/L citric acid, 10 g/L K_2_HPO_4_, 3.5 g/L NaNH_4_HPO_4_, 1 mM MgSO_4_, 0.1 mM CaCl_2_, pH 7.0) and Pseudomonas Isolation Agar with 50 μg/ml gentamicin (PIA Gm50; BD Difco) were used to isolate *P. aeruginosa* transconjugants. Tryptone Yeast 25% Sucrose (TYS25; 10 g/L tryptone, 5 g/L yeast extract, 25% w/v sucrose, 18 g/L agar) was used for SacB counterselection. Chemicals were purchased from MilliporeSigma (St. Louis, MO).

### Strains

*P. aeruginosa* strains used in this study were derivatives of *P. aeruginosa* strain PAO1. *S. enterica* serovar Typhimurium LT2 strains were available in the laboratory strain collection. Strains, plasmids, and primers used are listed in Table 2 and Table 3. *P. aeruginosa* deletion mutants were generated by allelic exchange as described previously [47]. In short, ∼800 bp upstream and downstream of the gene of interest was amplified by PCR. The DNA product was cloned by standard procedures into pMQ75. Assembled plasmids were then transformed into S17-1 *E. coli* by chemical transformation. Vectors were mobilized into *P. aeruginosa* by conjugation with S17-1 *E. coli* harboring vectors with homologous regions upstream and downstream of the gene of interest. Merodiploids, generated by homologous recombination of the plasmid into the chromosome, were isolated on VBMM Gm, then streaked for isolation on PIA Gm. SacB/sucrose counterselection at 30°C was used to select for excision of the plasmid from the chromosome via a second homologous recombination event. Sucrose resistant, gentamicin sensitive colonies were screened by PCR and deletions were confirmed by Sanger sequencing. Mini-Tn7 insertions were generated according to Schweizer *et al.* [48]. Briefly, pUC18T-mini-Tn7T-Gm and pTNS3 were transformed into *E. coli* S17-1 and S17-1 *pir+* respectively. Plasmids were mobilized simultaneously into *P. aeruginosa* strains by conjugation. Transconjugants were isolated on VBMM Gm50 and streaked for isolation on PIA Gm50. Drug resistant colonies were screened for insertion into the *att*Tn7 site by PCR with primers glmS-down and glmS-up.

**Table 2:** Plasmids and strains used in this study.

**Plasmid name**	**Description**	**Source**
pTNS3	Tn7 helper plasmid	[48]
pUC18T-mini-Tn7T-Gm	Delivery plasmid for Gm^R^ on Tn7	[48]
pMQ75	Gram-negative allelic replacement vector.	[53]
pMQ75-Δ*dadA*	pMQ75 with 800 bp of homology upstream and downstream of *dadA*	This study
pMQ75-Δ*dadY*	pMQ75 with 800 bp of homology upstream and downstream of *dadY*	This study
pCV1	pCV1 – empty vector	[51]
pDM1580	pCV1 – *P. aeruginosa* DadY	Laboratory collection
pDM1636	pCV1 – *S. enterica* RidA	[31]
pET28b(+)	BspQI modified pET28b(+)	[54]
pDM1652	pET28b(+) – *P. aeruginosa* DadA	This study
pDM1653	pET28b(+) – *P. aeruginosa* DadY	This study
**Strain ID**	**Genotype**	**Source**
*P. aeruginosa*		
DMPA1	Δ*dadA*	This study
DMPA4	MPAO1 wild-type (Manoil laboratory)	[55]
DMPA24	Δ*dadY*	This study
DMPA39	Δ*dadA att*Tn7::Gm^R^	This study
DMPA40	Δ*dadY att*Tn7::Gm^R^	This study
*S. enterica*		
DM14847	*ridA1*::Tn*10d* (Tc)/pCV1	Laboratory collection
DM16390	*ridA1*::Tn*10d* (Tc)/pDM1580	Laboratory collection
DM17142	*ridA1*::Tn*10d* (Tc)/pDM1636	Laboratory collection

All *P. aeruginosa* strains were derivatives of the MPAO1 wild-type strain (DMPA4).

**Table 3:** Primers used in this study.

**Primer**	**Sequence**
pae-MQ75-dadA-USF	cgggtaccgagctcgGCGCGGCGTGCCGAATACCAGA
pae-MQ75-dadA-USR	cgcgccgcgtgcggctGCAGTTGCGCAGCATCTGCAGCA
pae-MQ75-dadA-DSF	agccgcacgcggcgcgCCGACCTCTATCCCGAGGGC
pae-MQ75-dadA-DSR	ctatgaccatgattacgGGGCCAGCCGATAGTTGTGAC
dadA-seq-F	ATGCGAGTTCTGGTCCTTGG
dadA-seq-R	GTGTGCTGGCGCTGGATG
pae-MQ75-dadY-USF	atccccgggtaccgagctcgATGCTGCGCAACTGCACC
pae-MQ75-dadY-USR	ggcttcgacgGCCCGCGGACATGTCCTC
pae-MQ75-dadY-DSF	gtccgcgggcCGTCGAAGCCAAGCTCTG
pae-MQ75-dadY-DSR	cagctatgaccatgattacgGCATGTCCATCGATACCC
dadY-seq-F	GCAACTGCTCAAGCCGCTGGG
dadY-seq-R	AAGTCCTCGGGGAAGAAGCCGACG
dadA-pET-F	nngctcttcnATGCGAGTTCTGGTCCTTGG
dadA-pET-R	nngctcttcnGTGGTGTGCTGGCGCTGGA
dadY-pET-F	nngctcttcnATGCCCATCCAGCGCCAGCACA
dadY-pET-R	nngctcttcnGTGGGGCAGCGCAGCGACCACCG
glmS-down	GCACATCGGCGACGTGCTCTC

### Bioinformatics analyses

Sequences were obtained from the Pseudomonas Genome Database (PGBD) [49]. A DIAMOND BLASTP search was performed, using default parameters, with DadA from *P. aeruginosa* PAO1 as the query against all complete genomes in the PGBD [35]. Each species was sorted into one of five groups based on the composition of the *dad* operon. The groups and composition of the operon are as follows: Group 1, *dadRAX*; Group 2, *dadRAY*; Group 3, *dadRA*; Groups 4 and 5, *dadRAYX*. Groups 4 and 5 were distinguished based on the location of the gene encoding AsnC-type regulator DadR in relation to the rest of the operon – Group 5 *dadR* is three reading frames upstream of the *dadA* promotor. For a species to be placed in one of these groups, the majority of strains (compliance >60%) must have had the same operon composition. The groups of *Pseudomonas* species based on the structure of their *dad* operon was then compared to their phylogenetic classification [39, 41, 42, 50]. The phylogenetic groups and subgroups of *Pseudomonas* species were then classified based on the structure of the *dad* operon as described above, except that type strains of species within the group were used in leu of strains within a species, at a compliance threshold of >75%.

### Molecular techniques

Plasmids were constructed using standard procedures. Restriction endonucleases were purchased from New England Biosciences. Plasmids were isolated using the GeneJET Plasmid Miniprep Kit (Thermo Scientific). Q5 DNA polymerase (New England Biosciences) was used to amplify DNA and primers were synthesized by Eton Bioscience. PCR products were purified using the QIAquick PCR purification kit (Qiagen) and Sanger sequencing was performed by Eurofins Genomics. All DNA products were amplified by PCR using *P. aeruginosa* PAO1 gDNA as a template.

Homologous regions (∼800 bp) flanking *dadA* were amplified using the primer pair pae-MQ75-dadA-USF/pae-MQ75-dadA-USR for the upstream region, and primers pae-MQ75-dadA-DSF/pae-MQ75-dadA-DSR for the downstream region. Primers pae-MQ75-dadY-USF/pae-MQ75-dadY-USR were used to amplify the region 800 bp upstream of *dadY*, and pae-MQ75-dadY-DSF/pae-MQ75-dadY-DSR were used for the downstream region. Flanking regions for each respective gene were cloned into the EcoRI site of pMQ75. Sequencing primers dadA-seq-F/dadA-seq-R were used to confirm deletion of *dadA* and primers dadY-seq-F/dadY-seq-R were used to confirm deletion of *dadY*. The coding regions of *dadA* and *dadY* were amplified using primer pairs dadA-pET-F/dadA-pET-R and dadY-pET-F/dadY-pET-R respectively, and were cloned into the BspQI site of BspQI modified pET28b(+) [51]. Primers glmS-down/glmS-up were used to confirm insertion into the *att*Tn7 site of *P. aeruginosa* [48].

### Growth analysis

Growth (OD_650_) was monitored at 37°C in a 96-well plate using a BioTek ELx808 plate reader with a slow shaking speed. Overnight cultures (1 ml) in rich medium were grown at 37°C with shaking, pelleted and resuspended in an equal volume of sterile saline. Cell suspensions (5 μl) were used to inoculate each indicated medium (195 μl). Data were plotted using GraphPad Prism version 8.0.

### Protein purification

Three 10 ml cultures of *E. coli* BL21-AI harboring pDM1652 (DadA) or pDM1653 (DadY) were grown overnight at 37°C in TB Km (50 μg/ml) and used to inoculate each of three 2.8 L Fernbach flasks containing 1.5 L of TB Km. Cultures were grown at 37°C with shaking at 150 rpm. When the OD_650_ reached 0.6, cells were induced with 0.2% arabinose and 0.1 mM IPTG. The temperature was lowered to 23°C, and cultures were incubated for 18 h before being harvested by centrifugation. All purified proteins were flash-frozen in liquid nitrogen and stored at −80°C until use.

#### DadY

DadY was purified to a final concentration of 924 μM (12 mg/ml) and a purity of >95%. Cells were resuspended in 2 ml/g cell weight of Binding Buffer A (50 mM potassium phosphate pH 7.4, 150 mM NaCl, 20 mM imidazole) with lysozyme (2 mg/ml) and DNase (0.125 mg/ml). Cell suspensions were incubated on ice for 20 minutes before being mechanically lysed using a Constant Systems Limited One Shot (United Kingdom) at 20 kpsi. PMSF (1 mM) was added to the lysate before centrifugation at 45,000 x *g* for 45 min. Clarified cell lysate was filtered through a PVDF filter (0.45 μm pore size) before being loaded onto a 5 ml HisTrap HP Ni-Sepharose column, which was then washed with five column volumes of Binding Buffer A followed by four column volumes of 4% Elution Buffer A (50 mM potassium phosphate pH 7.4, 150 mM NaCl, 500 mM imidazole). Protein was eluted with a gradient of Elution Buffer A from 4% to 100% over 10 column volumes. Purified protein was concentrated using a centrifugal filer with a molecular weight cutoff of 10 kDa and moved into Storage Buffer A (50 mM Tris-HCl pH 7.5, 150 mM NaCl, 10% glycerol) using a PD10 column (GE Healthcare).

#### DadA

DadA was purified to a final concentration of 92 μM (4.3 mg/ml) and a purity of ∼82%. Cells were resuspended in 2 ml/g cell weight of Binding Buffer B (50 mM potassium phosphate pH 7.4, 500 mM NaCl, 20 mM imidazole, 0.1% Triton X-100) with lysozyme (2 mg/ml), DNase (0.125 mg/ml), and FAD (50 μM) and incubated on ice for one hour. Cells were then lysed mechanically at 33 kpsi. PMSF was added to the cell lysate, which was then clarified by centrifugation at 40,000 x *g* for 30 min and passed through a 0.45 μm PVDF filter. Cell free extract was loaded onto a 5 ml HisTrap HP Ni-Sepharose column, which was then washed with five column volumes of Binding Buffer B followed by four column volumes of 25% Elution Buffer B (50 mM potassium phosphate pH 7.4, 500 mM NaCl, 500 mM imidazole, 0.1% Triton X-100). Protein was eluted with a gradient of Elution Buffer B from 25% to 100% over 15 column volumes. Purified protein was concentrated using a centrifugal filer with a molecular weight cutoff of 30 kDa and moved into Storage Buffer B (50 mM Tris-HCl pH 7.5, 500 mM NaCl, 0.1% Triton X-100, 10% glycerol) using a PD10 column (GE Healthcare).

### Enzyme assays

All enzyme assays were performed at room temperature (25°C).

#### Imine deaminase activity of DadY

L-amino acid oxidase (LOX) assays were performed as described previously [21, 45]. In short, LOX was used to generate imine intermediates from L-amino acid substrates. Imines were derivatized with semicarbazide, generating semicarbazone compounds that were detected spectrophotometrically at an absorbance of 248 nm. The reaction mixture, at a final volume of 100 μl, contained potassium pyrophosphate pH 8.7 (50 mM), semicarbazide pH 7.0 (10 mM), bovine liver catalase (24 U), and L-amino acid oxidase from *Crotalus adamanteus* (1 μg). Assay mixtures contained 25 μM DadY or RidA where indicated. Reactions were initiated by the addition of the indicated substrate (20 mM) and absorbance at 248 nm was monitored for five minutes. The rate of semicarbazone formation was determined in the linear range for each assay mixture using the measured pathlength of each well and the extinction coefficient for semicarbazone (ε = 10300 M^-1^ cm^-1^).

#### Imine generation by DadA

Imine generation and release by DadA were assayed using a modified version of a previously described assay [21]. Briefly, assay mixtures (100 μl) contained potassium pyrophosphate pH 8.7 (50 mM), neutralized semicarbazide (10 mM), FAD (30 μM), ubiquinone-1 (100 μM), DadA (0.5 μM), and DadY (5 μM) where indicated. Reactions were initiated with 7.5 mM D-alanine and absorbance monitored at 248 nm for five minutes. The rate of semicarbazone formation was determined as described above for the LOX assay.

### Competition assays

Competition assays were performed as described previously [16, 52]. In short, overnight cultures (3 ml) of each strain were grown in LB at 37°C. Overnights were diluted to an OD_650_ of 0.1 in sterile saline and 50 μl of DMPA39 or DMPA40 were co-inoculated with an equal amount of DMPA4 in 5 ml of the indicated medium. Colony forming units (CFU) of the inoculum was determined by serial dilution in saline and spot plating onto LB to determine the total number of cells, and on LB with 30 μg/ml gentamicin (LB Gm30) to determine the number of DMPA39 or DMPA40. The number of wild-type cells was determined by subtracting the number of CFU on LB Gm30 from those on LB. As a control, fitness of DMPA39 and DMPA40 were measured in competition with DMPA24 and DMPA1, respectively. Co-cultures were incubated at 37°C with shaking until cultures reached stationary phase. Co-cultures were serially diluted in saline and plated on LB and LB Gm30 as described above. GraphPad Prism version 8.0 was used for data plotting and statistical analysis.

**Figure:**
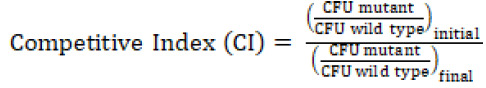


## SUPPLEMENTAL MATERIAL

Click here for supplemental data file.

All supplemental data for this article are available online at www.microbialcell.com.

## Abbreviations:

LOX: – L-amino acid oxidase.
