# Supplementary material for: DadY (PA5303) is required for fitness of Pseudomonas aeruginosa when growth is dependent on alanine catabolism
Source: Microb Cell. 2022 Nov 22;9(12):195–206. doi: 10.15698/mic2022.12.788 (PMC9714295; doi:10.15698/mic2022.12.788)
Supplement: Supplementary file 1 [file mic-09-195-s01.pdf]

SUPPLEMENTAL INFORMATION

DadY (PA5303) is required for fitness of *Pseudomonas aeruginosa* when growth is dependent on alanine catabolism

Ronnie L. Fulton, and Diana M. Downs*

Department of Microbiology

University of Georgia

Athens, GA 30602-2605

S

Protein	DadA	DadY	SeRidA	PaRidA
approx. MW	47 kDa	13 kDa	13 kDa	13.5 kDa
Purity	82%	99%	95%	99%

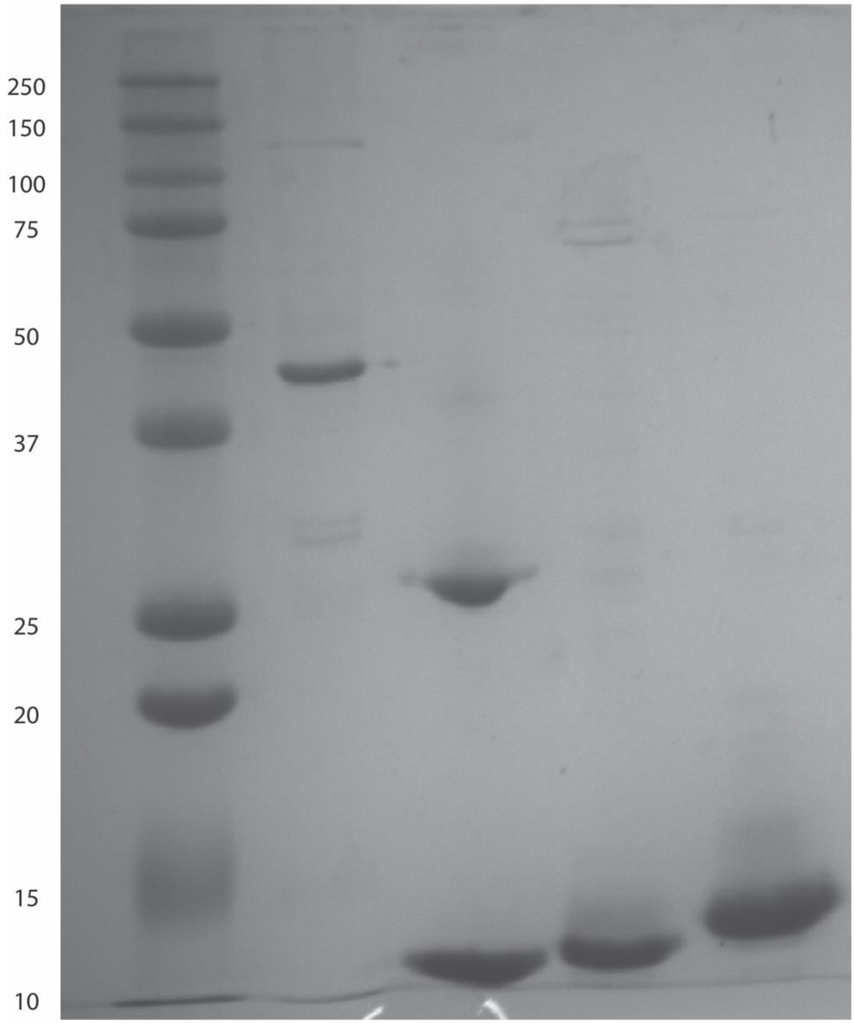

Figure S1: Purified DadA, DadY, $_{SE}$RidA and $_{PA}$RidA. Proteins were purified from *E. coli* BL21-AI. Samples were boiled in buffer containing β-mercaptoethanol and ~4 μg of each sample was loaded onto a 12% polyacrylamide gel before being separated by electrophoresis. The gel was stained with Coomassie Blue and imaged using AnalytikJena UVP ChemStudio. Purity of each sample was determined by densitometry using VisionWorks software version 8.22.18309.10577. The 26 kDa band in the DadY sample was confirmed to be DadY dimer by Peptide Mass

Fingerprinting analysis at the University of Georgia Proteomics Core. BioRad Precision Plus. Protein Dual Color Standards were run in the left-most lane.
